# Multilevel polarization switching in ferroelectric thin films

**DOI:** 10.1038/s41467-022-30823-5

**Published:** 2022-06-07

**Authors:** Martin F. Sarott, Marta D. Rossell, Manfred Fiebig, Morgan Trassin

**Affiliations:** 1 Department of Materials, ETH Zurich, CH-8093 Zurich, Switzerland; 2 Electron Microscopy Center, Empa Swiss Federal Laboratories for Materials Science and Technology, CH-8600 Duebendorf, Switzerland

**Keywords:** Ferroelectrics and multiferroics, Nonlinear optics

## Abstract

Ferroic order is characterized by hystereses with two remanent states and therefore inherently binary. The increasing interest in materials showing non-discrete responses, however, calls for a paradigm shift towards continuously tunable remanent ferroic states. Device integration for oxide nanoelectronics furthermore requires this tunability at the nanoscale. Here we demonstrate that we can arbitrarily set the remanent ferroelectric polarization at nanometric dimensions. We accomplish this in ultrathin epitaxial PbZr_0.52_Ti_0.48_O_3_ films featuring a dense pattern of decoupled nanometric 180° domains with a broad coercive-field distribution. This multilevel switching is achieved by driving the system towards the instability at the morphotropic phase boundary. The phase competition near this boundary in combination with epitaxial strain increases the responsiveness to external stimuli and unlocks new degrees of freedom to nano-control the polarization. We highlight the technological benefits of non-binary switching by demonstrating a quasi-continuous tunability of the non-linear optical response and of tunnel electroresistance.

## Abstract (web summary)

Setting any polarization value in ferroelectric thin films is a key step for their implementation in neuromorphic devices. Here, the authors demonstrate continuous modulation of the remanent polarization at the nanoscale in PbZr_0.52_Ti_0.48_O_3_ films.

## Introduction

The switchable bistable polarization in ferroelectrics allows for the binary control of optical^1^, electronic^2^, and catalytic properties^3^ that are essential for a wide range of applications. Going beyond the limitation of a binary remanent polarization, however, could hold great promise for the development of novel nanoelectronic devices^4–6^. The multilevel polarization switching at the nanoscale required for this may be conveniently achieved through systems, where small external perturbations lead to pronounced, often non-linear responses. This requirement is, for instance, met when approaching phase boundaries at which a structural competition flattens the thermodynamic energy landscape^7–10^ and renders the system intrinsically non-linear. A prime example of this can be found in the most technologically relevant ferroelectric PbZr_1−*x*_Ti_*x*_O_3_ (PZT) family^11^. By precisely controlling the B-site occupancy, a structural competition is obtained at the so-called morphotropic phase boundary (MPB) which separates the tetragonal from the rhombohedral phase. The resulting high susceptibility is a main reason for the outstanding technological relevance of PZT.

For emerging applications, continuous tunability of the remanent response is required, and for this, having a handle to control the response of the system to external stimuli is vital. In ferroelectrics, based on the strong intrinsic relation between lattice and polarization, epitaxial strain in thin films represents a powerful tool to accomplish that and thus tailor the electric-field response. For instance, imposing epitaxial strain has been extensively used to enhance the polarization magnitude^12^, increase the Curie temperature *T*_C_^13,14^, manipulate the domain configuration^15–17^, act on the switching mechanism^18^, and even trigger structural instabilities^19,20^. The benefits of epitaxial strain are fully exploited in the ultrathin regime, where the effect of strain relaxation is still negligible.

Thus, we propose that the right combination of the inherently increased susceptibility in the vicinity of the MPB and control brought about by epitaxial strain is key for the design of a ferroelectric response that is continuously tunable at the nanoscale. The influence of residual strain on dielectric properties at the MPB has been investigated in rather thick PZT films^21^, but until now, there have been no reports where a continuous nano-control of the remanent polarization was accomplished.

Here, we show that we can arbitrarily tune the remanent net polarization of ultrathin PZT films at the MPB under compressive epitaxial strain and at the nanoscale via local application of a DC voltage. The resulting multilevel polarization switching is mediated by strain-stabilized nanoscale domains that exhibit a broad distribution of coercive field values. Using a combination of in-situ second harmonic generation (ISHG), piezoresponse force microscopy (PFM), and X-ray diffraction (XRD), we track the formation of nanometric 180° domains within the ferroelectric system. The observed switching characteristics allow for a quasi-continuous tuning of functionalities coupling to the electric polarization, such as optical second harmonic generation (SHG), which is a key ingredient of photonic technologies^22–24^. We furthermore demonstrate variable electroresistance through a ferroelectric tunnel barrier, which can be of great significance for memristive applications.

## Results

We begin our investigation by monitoring the emergence of the ferroelectric polarization *P* during the epitaxial growth of PbZr_0.52_Ti_0.48_O_3_ films with a nominal composition near the MPB (hereafter referred to as PZT_MPB_) on conducting SrRuO_3_-(SRO)-buffered (110)-oriented NdScO_3_ (NSO). To access *P* and follow its evolution during the pulsed laser deposition process, we use ISHG^14^. Optical SHG denotes frequency doubling of light in non-centrosymmetric media. In ferroelectrics, ordering of electric dipoles at *T*_C_ breaks inversion symmetry and gives way to SHG with an intensity *I*_2*ω*_ ∝ *P*^2^ ^25,26^. Here, the compressive epitaxial strain of −1.1% raises *T*_C_ above the growth temperature of 550 °C and stabilizes the SHG-active ferroelectric phase of PZT_MPB_ during growth. Complementary to ISHG, we use reflection high-energy electron diffraction to monitor the film thickness with unit-cell accuracy (Fig. S1).

**Fig. 1:**
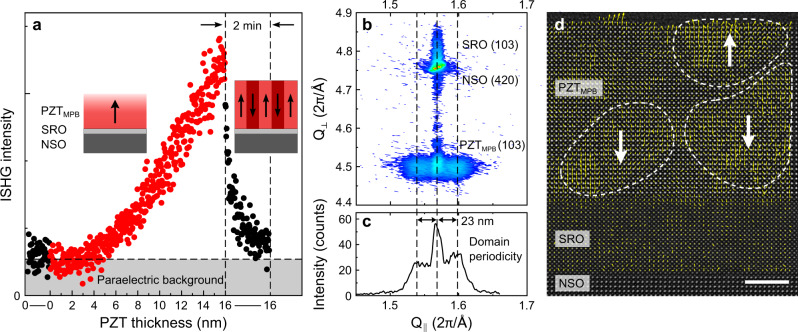
Formation of nanoscale 180° domains in strained PZT_MPB_ thin films. **a** ISHG signal evolution during the ongoing growth of PZT_MPB_ on SRO-buffered NSO (red symbols) and at halted growth (black symbols). The insets illustrate the prevailing domain configurations during and after growth. **b** Reciprocal space map (out-of plane *Q*_⊥_ vs. in-plane *Q*_||_) around NSO 420 and PZT_MPB_ 103. The PZT_MPB_ film is fully strained with an extracted tetragonality *c*/*a* of 1.04. The dashed vertical lines indicate the main peak and satellite peak positions. **c** Cross-section at fixed *Q*_⊥_ across the intensity distribution around the PZT_MPB_ 103 reflection. **d** HAADF-STEM image with overlaid ferroelectric dipole map viewed along the [010] zone axis. The yellow arrows reveal the presence of oppositely polarized 180° domains delimited by the dashed white lines. The white arrows represent the net polarization of each nanodomain. Scale bar, 4 nm.

The ISHG intensity as a function of the PZT_MPB_ thickness is shown in Fig. 1a. A signal onset above the paraelectric background, corresponding to an out-of-plane polarization component, is evident after the deposition of 4 nm of PZT_MPB_. The subsequent, continuous ISHG increase is consistent with the establishment of an out-of-plane-oriented spontaneous polarization driven by compressive strain^17^ with a single-domain configuration (Fig. S2) stabilized by the charge-screening environment of the conducting SRO layer and the growth atmosphere.

After halting the growth, however, the ISHG signal decays to the paraelectric level within 2 min, indicating quenching of the net out-of-plane polarization *P*_net_. This quench can be the result of suppression of electric dipole moments^27^, rotation of *P*_net_ away from the out-of-plane direction, or a ±*P* multidomain breakdown towards *P*_net_ = 0. In a multidomain state, the destructive interference of SHG waves emitted from oppositely polarized domains can suppress the net SHG signal^17,25^.

To clarify which of the above options applies, we perform ex-situ XRD experiments at room temperature. The reciprocal space map in Fig. 1b reveals purely tetragonal PZT_MPB_, fully in-plane-strained to the NSO substrate. The presence of broad satellite peaks in the diffuse scattering around the PZT_MPB_ 103 (Fig. 1c) and 002 (Fig. S3) reflections indicates a repetitive structure with an in-plane periodicity of 23 nm. Satellite peaks are frequently observed upon the formation of a regular distribution of nanoscale 180° domains^28^. Here, the in-plane scattering (Fig. S3) points to a characteristic maze domain pattern consisting of out-of-plane polarized 180° domains with a preferred orientation along the [1$\bar{1}$0] direction of the substrate. The high-angle annular dark-field scanning transmission electron microscopy (HAADF-STEM) image with electric dipole mapping in Fig. 1d confirms the presence of oppositely polarized nanoscale regions with a lateral size on the order of 10 nm. This domain formation in our coherently strained films is rather surprising, since the presence of a charge-screening bottom electrode reduces the depolarizing field that typically drives the formation of 180° domains in the ultrathin regime^29^. Hence, thin films made from well-known ferroelectrics like PZT, PbTiO_3_, or BiFeO_3_ exhibit a uniformly out-of-plane polarized single-domain configuration on a bottom electrode. Here, however, the Pb-rich top surface of Pb-containing films results in an additional surface-charge contribution^27,30^ that destabilizes the polarization and triggers domain formation in our highly electric-field-sensitive films near the morphotropic phase boundary.

**Fig. 2:**
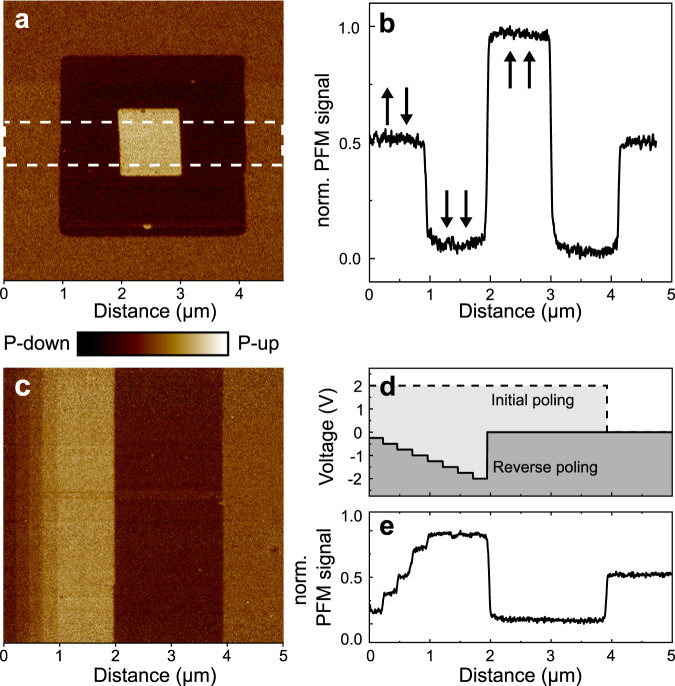
Local switching characteristics of the PZT_MPB_ thin films. **a** The vPFM image after locally applying +4 V (outer square) and −2 V (inner square) between the scanning tip and the SRO bottom electrode. **b** Averaged vPFM signal across the dashed outline in (**a**). **c** The vPFM image after applying the incremental reverse poling scheme detailed in (**d**). **d** Poling voltage as function of the sample location for the two-step poling scheme applied on the image area in (**c**). **e** Averaged horizontal vPFM cross-section over the entire image in (**c**).

To investigate the response of the nanoscale domain configuration to external electric fields, we move to PFM. Figure 2a shows the vertical-PFM (vPFM) image obtained after applying a positive or negative voltage between the tip and the SRO bottom electrode to a box-in-box region on the sample. The averaged PFM signal across the marked region shown in Fig. 2b clearly exhibits three distinct levels, indicating that the poling has converted the film into a fully up- or down-polarized state, whereas the pristine film is in a multidomain configuration with an equal fraction of both of these domain states. The homogeneous vPFM response of the pristine film corroborates the existence of domains with lateral size below the PFM resolution limit in agreement with the estimated sub-30-nm extension of the domains derived from XRD (Fig. 1b) and STEM (Fig. 1d).

Surprisingly, when gradually reversing the electric field applied to a polarization-saturated region (poling scheme in Fig. 2d), each voltage increment translates into a discrete and homogeneous step in the remanent vPFM signal until the opposite polarization-saturated state is reached, as shown in Fig. 2c, e. The stability of the PFM response after waiting times of up to several months (Fig. S4) excludes measurement artefacts, charging effects, or surface adsorbates as origin of the PFM signal^31^. For arbitrary selected polarization levels, we observe a distinct and stable PFM response for up to at least 10^4^ electric-field cycles (Fig. S5). Most strikingly, an appropriate value of the applied voltage even allows to locally restore the PFM signal characterizing the pristine nanodomain state with zero net polarization. To the best of our knowledge, this is the first demonstration of the local reversible formation of a remanent nano-sized domain configuration with *P*_net_ = 0 by one-time application of a DC voltage.

The ability to reach an arbitrary remanent polarization in Fig. 2c points to a mechanism that considerably differs from conventional ferroelectric poling, which is governed by abrupt nucleation and subsequent growth of oppositely polarized macroscopic domains when the applied electric field locally exceeds the coercive field (Fig. S6)^32^. In stark contrast, the domains in our samples retain their original nanoscopic size during poling. For each poling increment, the change of the vPFM signal in Fig. 2c indicates a shift in the population of these sub-resolution domains. We verified this switching behavior on 10 samples and note that it can only be observed for films near the MPB and in a specific coherently strained state (Fig. S6). The flattened energy landscape of the PZT_MPB_ film promotes the existence of a broad coercive field distribution, which is likely caused by inhomogeneous strain^33,34^ exerted by the NSO substrate with its anisotropic pseudocubic in-plane lattice constants (−0.97% vs. −1.28% strain). This decoupled nanometric domain configuration shows intriguing similarities to the polar nanoregions (2–10 nm) of relaxor-type ferroelectrics^35–37^. In relaxors, the polar clusters are often separated by a non-polar matrix, while in our films this decoupling is effectively realized by the lateral coercive field distribution. Furthermore, both relaxor ferroelectrics and ferroelectrics with a morphotropic phase boundary composition are well recognized as chemically disordered systems with an established high responsiveness to external stimuli. Aside from this analogy, the microscopy of relaxors and our PZT_MPB_ films are quite different, however. For example, in contrast to the time and frequency-dependent behavior of relaxors^38^, the polarization in our films reorients remanently without any noticeable relaxation after poling.

**Fig. 3:**
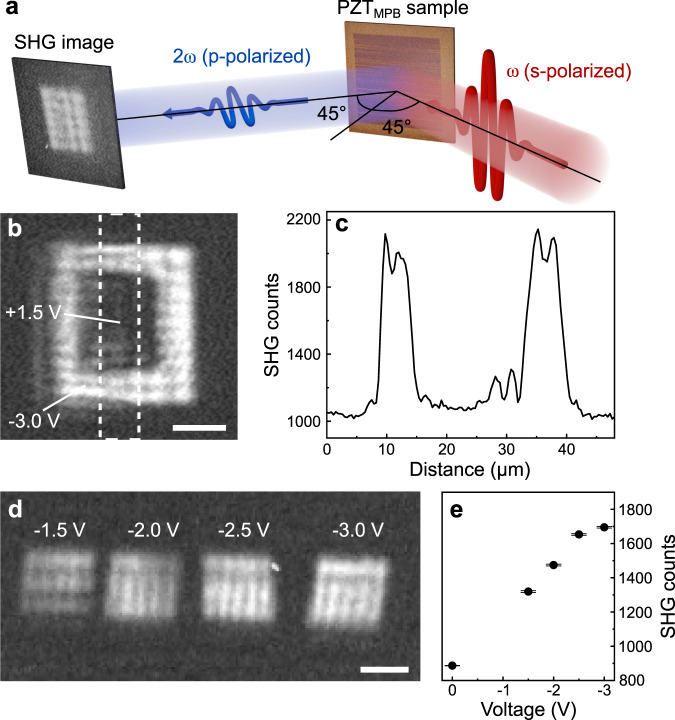
Continuous tunability of the SHG emission via DC voltage application. **a** Schematic SHG imaging setup in 45° reflection. **b** SHG image after applying −3.0/+1.5 V to a box-in-box region. The inner box shows the reversibly depolarized SHG-inactive state. **c** Cross-section of the SHG signal across the dashed outline in (**b**). **d** SHG image containing four neighboring 15 × 15 μm^2^ square areas, where the poling voltage is gradually increasing from left to right. Scale bars in **b**, **d** 10 μm. **e** Spatially averaged SHG signal for each poled area and the pristine background in **d** as a function of the applied voltage with indicated standard error.

This possibility to locally and reversibly modulate the remanent polarization continuously between depolarized and saturated furthermore provides a handle on inversion-symmetry-governed functionalities, simply by the application of the appropriate DC voltage. One such functionality, which is of particular relevance for photonic applications^22,23^, is SHG. The ability to generate and manipulate photons is indeed of high relevance for on-chip nanooptics^39^. To investigate our control of the local SHG response, we perform spatially resolved SHG microscopy as illustrated in Fig. 3a. Using the PFM tip, we first induce an upwards polarized 30 × 30 μm^2^ box. We then re-establish the depolarized state in an interior 15 × 15 μm^2^ box. The resulting SHG image in Fig. 3b reveals that the film is only SHG-active in the upwards polarized area, whereas for both the multidomain configuration of the pristine area and the depolarized box no SHG emission is observed. The absence of SHG in the latter is caused by destructive interference of SHG waves emitted from oppositely polarized nanoscale domains^17^. Thus, as exemplified by the SHG cross-section in Fig. 3c, we can indeed activate and deactivate the SHG emission at will by controlling the population of upwards and downwards polarized nanometric domains. The perfect agreement between SHG imaging and PFM, despite the different probing depth of each technique, clearly points towards homogeneous switching throughout the film volume, rather than a surface-related phenomenon.

The SHG image in Fig. 3d shows four 15 × 15 μm^2^ regions poled next to one another with increasing voltage. Each area exhibits an average SHG signal that increases from left to right (Fig. 3e), because of the gradual change in the remanent polarization. Hence, these SHG microscopy experiments demonstrate the capacity in our PZT_MPB_ films to freely and reversibly tune the non-linear optical response on selected locations beyond the realm of binary on/off switching^40^ and even revert to the pristine SHG-inactive state.

For a second application exploiting the quasi-continuous variability of our nanoscale domain configuration, we move to ferroelectric tunnel junctions (FTJs). In FTJs, the tunnel current across a thin ferroelectric layer depends on the direction and the magnitude of the polarization^2,41^ and can be modulated by controlling the density of oppositely oriented domains at the sub-micrometer scale^4,42^. Thus, FTJs have attracted a lot of attention as basis for memristive devices^4^ and neuromorphic engineering^5^.

**Fig. 4:**
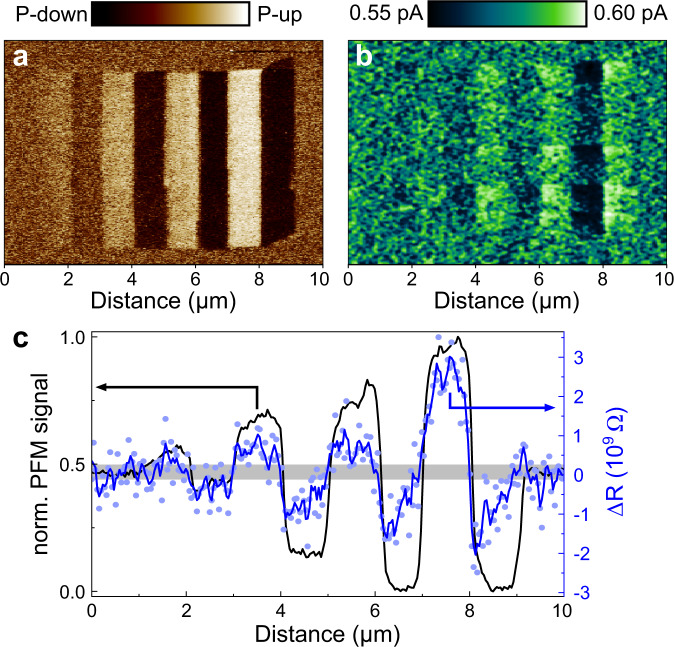
Variable electroresistance in ultrathin PZT_MPB_ films. **a** The vPFM image on a 4 nm PZT_MPB_ film displays a gradual polarization switching. **b** Conductive atomic force microscopy map on the same area as in **a** with a 100 mV DC reading voltage. **c** Averaged vPFM signal (black) and resistance change (blue) cross-sections across the poled stripes in **a**, **b**, respectively. The blue line shows the three-point moving average of the blue data points. The gray line serves as a guide to the eye to show the PFM signal and resistance of the as-grown state.

Figure 4a shows the vPFM image of a PZT_MPB_ film with a thickness of 10 unit cells after applying consecutively increasing voltages to the scanning tip on a succession of 5 × 1 μm^2^ stripes. The remanent PFM signal confirms the presence of the now-familiar nano-sized domain configuration with multilevel polarization switching in this ultrathin regime. On the nano-polarized stripes, we then measure the polarization-dependent current under a DC reading voltage of 100 mV, using conductive atomic force microscopy (Fig. 4b). As shown in the cross-section depicting the resistance change (Δ*R*) in Fig. 4c, the upwards polarized stripes exhibit an increased resistance, whereas a down polarization results in a resistance decrease with respect to the pristine state that is stable with time for more than 24 h (Fig. S7). Most importantly, we recognize a gradual change in resistance that scales with the magnitude of the remanent out-of-plane polarization. Thus, the PZT_MPB_ films presented here, exhibit a memristor-like behavior in FTJs with a broad tunability of the tunnel electroresistance TER = $({R}_{P\uparrow }-{R}_{P\downarrow })/{R}_{P\downarrow }$, where ${R}_{P\uparrow },{R}_{P\downarrow }$ denote the resistances of the fully upwards and downwards polarized regions, respectively. In our proof-of-concept experiment the TER at room temperature reaches 1.9%. This value further increases to 42.9% for 7 unit cell PZT_MPB_ layers. Unlike in conventional FTJs, where a distribution of domains with a lateral size above 100 nm sets the resistance state, the sub-30-nm size of the ferroelectric domains in our films enables multilevel polarization switching well below common device dimensions^4^, thus pushing device miniaturization to its fundamental limits. The strained PZT_MPB_ system could therefore provide a hitherto unexplored high-storage-density platform to emulate synaptic behavior for neuromorphic device paradigms down to the nanoscale.

## Discussion

In summary, we have demonstrated that the combination of the increased susceptibility at the MPB and anisotropic compressive strain induces the formation of a stable distribution of nanoscale ferroelectric 180° domains in PZT_MPB_ films. The decoupled nanometric domain configuration and its distribution of coercive fields persists upon poling and allows to locally tune the net polarization. Making use of this tunability, we demonstrate the continuous manipulation of inversion-symmetry-related functionalities with the continuous polarization control leading to variable SHG activity and electroresistance in FTJs at the nanoscale.

## Methods

### Sample preparation and structural characterization

The PZT_MPB_/SRO films were grown on single-crystalline NSO (110) substrates (CrysTec GmbH) with pulsed laser deposition using a 248 nm KrF excimer laser. The SRO layer was deposited at 700 °C under 0.015 mbar oxygen pressure with a laser fluence of 0.95 J cm^−2^ (2 Hz). The PZT_MPB_ layer was subsequently grown at 550 °C under 0.12 mbar oxygen pressure with a laser fluence of 1.2 J cm^−2^ (4 Hz). The film thickness was monitored during growth using reflection high-energy electron diffraction and was confirmed ex-situ with X-ray reflectivity. Topography and PFM measurements of the thin films were performed on a Bruker Multimode 8 atomic force microscope and an NTEGRA Prima scanning probe microscope (NT-MDT Spectrum Instruments). The vertical PFM data was acquired in Cartesian coordinates with the two lock-in amplifier outputs *X* (in-phase component) and *Y* (out-of-phase component) that relate to amplitude *R* and phase *ϕ* as: $X=R\cdot {\cos }(\phi )$ and $Y=R\cdot {\sin }(\phi )$. Using a periodically poled LiNO_3_ reference sample, the internal phase of the lock-in amplifier was adjusted to obtain the complete vPFM signal in the *X* channel with the *Y* channel containing only the error. The crystal structure was analyzed by X-ray diffraction and reciprocal space mapping using a four-cycle thin-film diffractometer operating with CuK_*α*1_ radiation (PanAnalytical X’Pert^3^ MRD).

### In situ second harmonic generation (ISHG)

The output of an amplified Ti:Sapphire laser system (wavelength: 800 nm, repetition rate: 1 kHz, pulse duration: 45 fs) is converted by an optical parametric amplifier into the fundamental incident light with a wavelength of 1200 nm. The incident light with a pulse energy of 20 μJ is then focused onto the sample inside the growth chamber with a focus diameter of 250 μm and an angle of incidence of 45°. The reflected SHG light at 600 nm is selected by a monochromator and detected with a photomultiplier system. Both the incident light and the detected SHG light are polarized parallel to the plane of reflection. To optimize the alignment, a reference beam obtained from SHG in the optical components is used. Before data acquisition, this reference beam is removed using optical filters. The residual background originates from surface SHG and is referred to as “paraelectric background” in Fig. 1a.

### Scanning transmission electron microscopy (STEM)

Cross-sectional specimens for transmission electron microscopy analysis were prepared by means of a FEI Helios NanoLab 600i focused-ion beam (FIB) operated at accelerating voltages of 30 and 5 kV. High-angle annular dark-field scanning transmission electron microscopy (HAADF-STEM) was carried out using a FEI Titan Themis microscope equipped with a CEOS DCOR spherical-aberration probe corrector operated at 300 kV. A probe semi-convergence angle set to 18 was used in combination with an annular semi-detection range of the HAADF detector to collect electrons between 66 and 200 mrad. The HAADF-STEM images were obtained by averaging time series of 10 frames, after nonrigid registration using the Smart Align software^43^. Subsequently, the atomic column positions in the averaged HAADF-STEM images were fitted with picometer precision by means of seven-parameter two-dimensional Gaussians using custom-developed MATLAB scripts as described in refs. ^44,45^. Ferroelectric dipole maps were calculated from the relative displacements of the two cation sublattices present in the PbZr_0.52_Ti_0.48_O_3_ structure. Thus, the local ferroelectric dipoles were calculated by measuring the polar displacement in the image plane of the Zr/Ti position from the center of mass of its four nearest Pb neighbors. Here, in the ferroelectric dipole map of Fig. 1d derived from the HAADF-STEM image, the electric dipoles are plotted opposite to the displacement of the Zr/Ti cations.

### Optical SHG microscopy

For the *4mm* point-group symmetry of our PZT_MPB_ films with the ferroelectric polarization along the out-of-plane-oriented *z*-axis the following SHG tensor components are allowed: ${\chi }_{{zzz}}^{(2)}$, ${\chi }_{{zyy}}^{(2)}={\chi }_{{zxx}}^{(2)}$, ${\chi }_{{yzy}}^{(2)}={\chi }_{{xzx}}^{(2)}={\chi }_{{yyz}}^{(2)}={\chi }_{{xxz}}^{(2)}$. To obtain a non-zero SHG signal from an out-of-plane polarization and to spatially resolve the SHG images, a reflection setup with a 45° angle of incidence was used. The incident light was polarized along the *y*-axis perpendicular to the plane of reflection (s-in) and the image was created by SHG light polarized parallel to the reflection plane (p-out) to selectively probe the ${\chi }_{{zyy}}^{(2)}$ tensor component. As for the ISHG experiments, we used incident fundamental light with a wavelength of 1200 nm and a pulse energy of 25 μJ. The generated SHG light was collected with a ×20 long-working-distance microscope objective and projected onto a liquid-nitrogen-cooled back illuminated chip of a CCD camera (Symphony II, Horiba Jobin Yvon GmbH). We used optical filters to suppress unwanted wavelength components. To account for the 45° reflection geometry, a trapezoidal perspective correction with the following transformation matrix *T* was was applied to all images with the software GIMP to revert the poled regions into a square shape.

$${T}=\left(\begin{array}{ccc}1.1540 & 0.0000 & 0.0000\\ 0.0043 & 0.7906 & 94.4167\\ 0.0006 & \,0.0000 & 1.0000\end{array}\right)$$

### Conductive atomic force microscopy

Spatially resolved current maps were measured using an NTEGRA Prima scanning probe microscope (NT-MDT Spectrum Instruments) equipped with Pt-coated Si tips (MikroMasch, <30 nm tip radius). The thin-film samples were fixed with silver paint onto metal disks and mounted on the bottom of the scanner to ensure mechanical stability. For poling, DC voltages ranging between +3.25 and −3.25 V were applied between the bottom SRO electrode and the tip. The current maps were measured under a small DC bias of +100 mV with a 10 fA precision using a current amplifier connected to the cantilever. For the current map shown in Fig. 4b a 3-point Gaussian filter was applied after acquisition.

### Reporting summary

Further information on research design is available in the Nature Research Reporting Summary linked to this article.

## Supplementary information

Supplementary Information

Lasing Reporting Summary

## Data Availability

The data that supports the findings of this study are available from the corresponding authors upon reasonable request.
