# Supplementary material for: Multilevel polarization switching in ferroelectric thin films
Source: Nat Commun. 2022 Jun 7;13:3159. doi: 10.1038/s41467-022-30823-5 (PMC9174202; doi:10.1038/s41467-022-30823-5)
Supplement: Supplementary file 1 — Supplementary Information [file 41467_2022_30823_MOESM1_ESM.pdf]

# Supplementary Information
# Multilevel polarization switching in ferroelectric thin films

Martin F. Sarott,[1,*] Marta D. Rossell,[2] Manfred Fiebig,[1] and Morgan Trassin[1,†]

[1]*Department of Materials, ETH Zurich, CH-8093 Zürich, Switzerland.*

[2]*Electron Microscopy Center, Empa Swiss Federal Laboratories for Materials Science and Technology, CH-8600 Dübendorf, Switzerland.*

[*] martin.sarott@mat.ethz.ch

[†] morgan.trassin@mat.ethz.ch

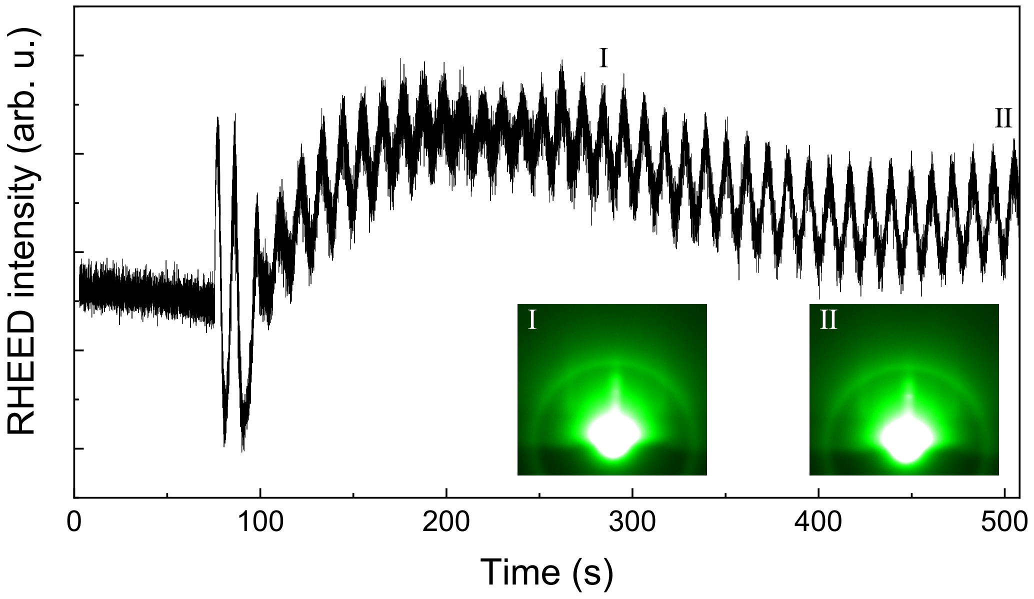


Figure S1. **RHEED intensity oscillations during the growth of $PZT_{MPB}$ on SRO-buffered NSO (110).** The insets show the unchanged RHEED patterns after the deposition of (I) 20 and (II) 40 unit cells, respectively.

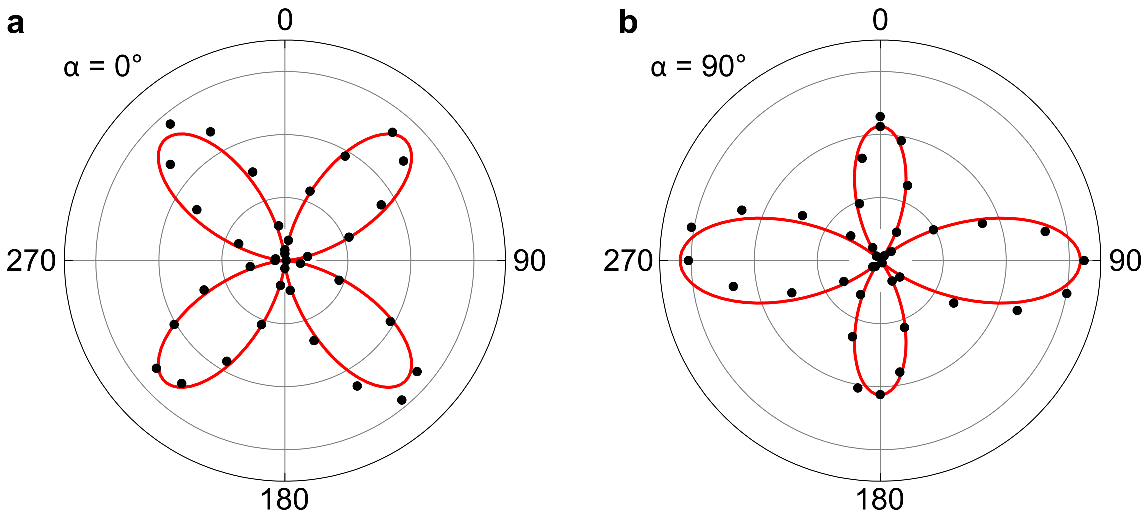


Figure S2. **SHG polarizer measurements for a** 45 nm **$PZT_{MPB}$ film on SRO-buffered NSO $(110)_o$.** In polarizer measurements, the incident fundamental light polarization is varied from 0° to 360° while keeping the detected SHG light polarization at a constant angle $\alpha$. **a,b** Polarizer scans at fixed analyzer angles of **a** $\alpha = 0°$ (s-out) and **b** $\alpha = 90°$ (p-out). The fit was calculated according to the procedure described in [1] for the $4mm$ point group.

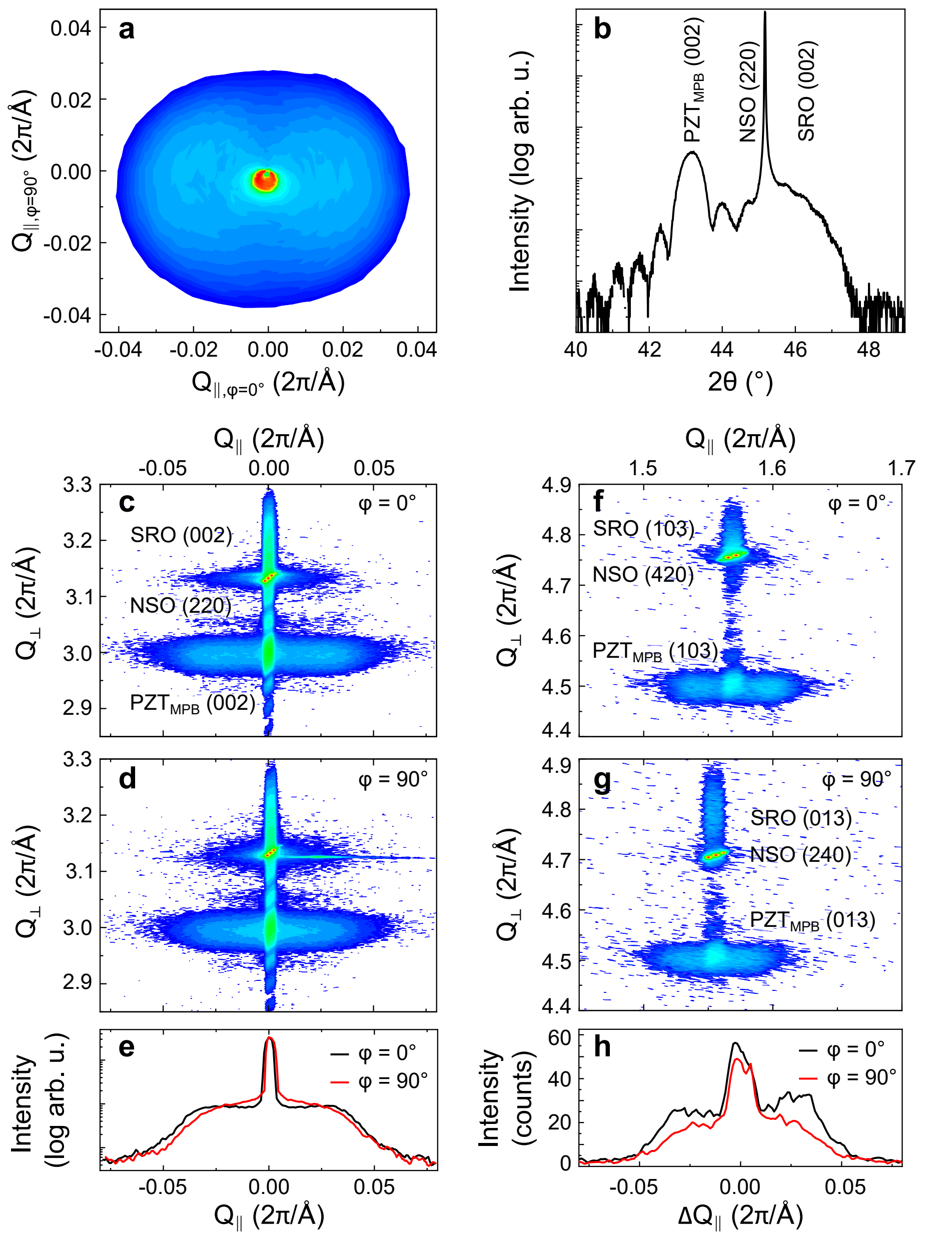


Figure S3. **XRD measurements of the $PZT_{MPB}$ films revealing the ordering of the 180° polarization nanodomains.** **a** In-plane reciprocal space map around the $PZT_{MPB}$ 002 peak. **b** Symmetric Θ-2Θ scan near the NSO 220 reflection displaying Laue oscillations around the $PZT_{MPB}$ 002 peak. **c,d** Reciprocal space maps around NSO 220 and $PZT_{MPB}$ 002 for two orthogonal in-plane directions with an azimuthal angle of **c** $\phi = 0°$ and **d** $\phi = 90°$, respectively. **e** Line profiles across the $PZT_{MPB}$ 002 reflection in **c,d**. **f,g** Reciprocal space maps around **f** NSO 420 and **g** NSO 240, respectively. **h** Line profiles across the $PZT_{MPB}$ 103 and 013 reflections in **f,g**. The intensity maxima of the diffuse in-plane scattering appear at an equal distance from the main peak for both orthogonal scan directions, indicating the same domain periodicity along both directions.

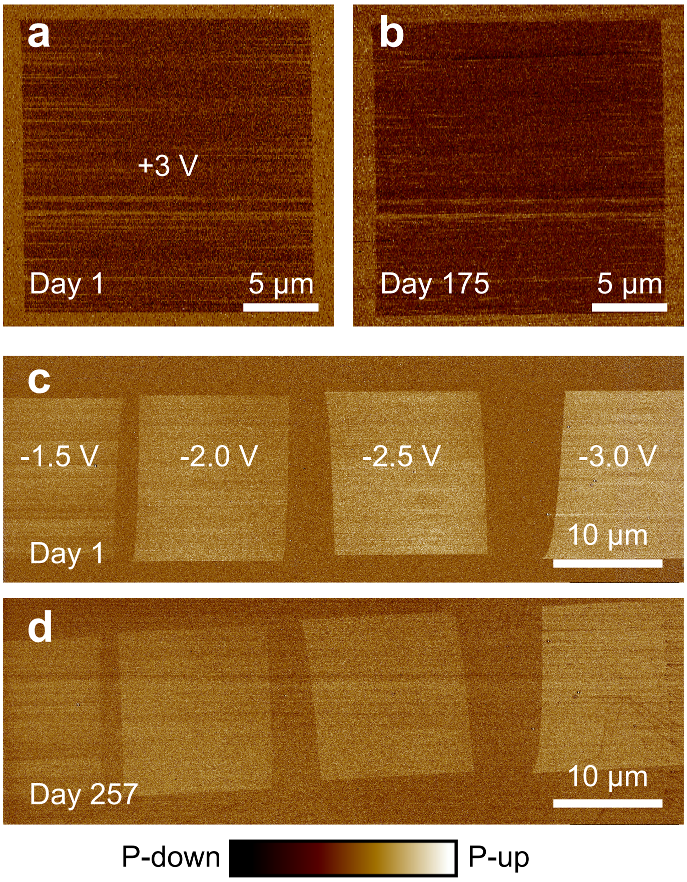


Figure S4. **Temporal stability of the electric-field-induced polarization switching. a-d** vPFM images acquired directly after poling and after extended periods of time for **a**, **b** a $20\times20\,\mu\mathrm{m}^2$ downwards poled box and **c**, **d** a series of four $15\times15\,\mu\mathrm{m}^2$ increasingly upwards poled regions from left to right.

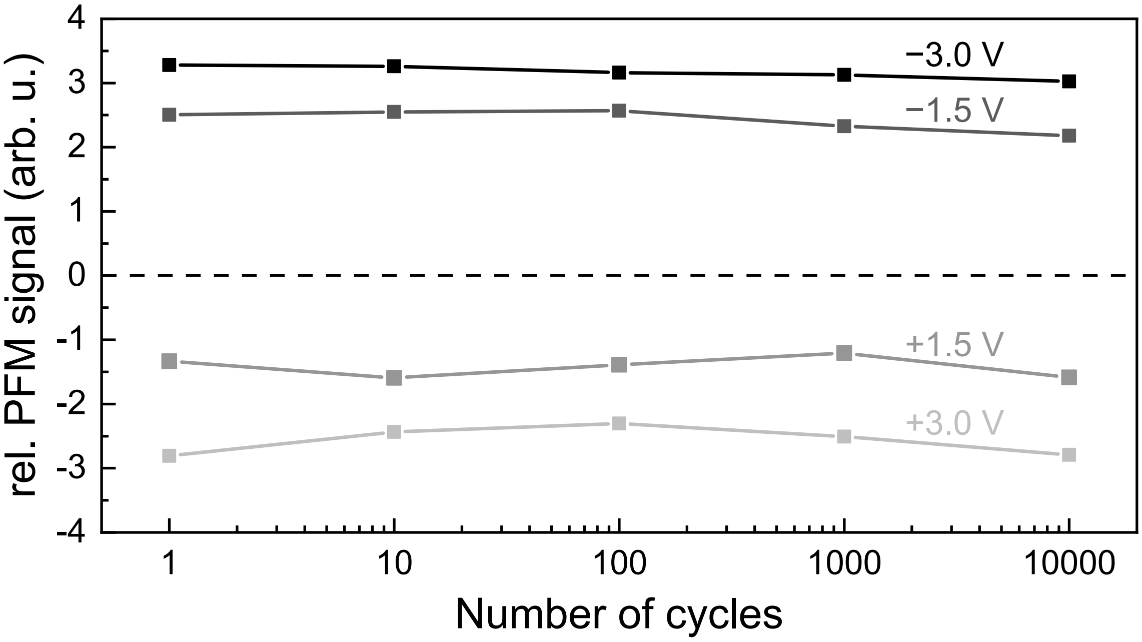


Figure S5. **Endurance measurement of the remanent vPFM signal for four different levels of $P_{\mathrm{net}}$ with respect to the as-grown multidomain configuration.** For electric field cycling, square voltage pulses were applied to a static PFM tip at 100 Hz using an arbitrary function generator (Tektronix AFG2021). The vPFM signal at remanence was subsequently measured at the location of the scanning tip. The endurance measurement reveals a clear retention of all four levels of $P_{\mathrm{net}}$ for at least $10^4$ cycles.

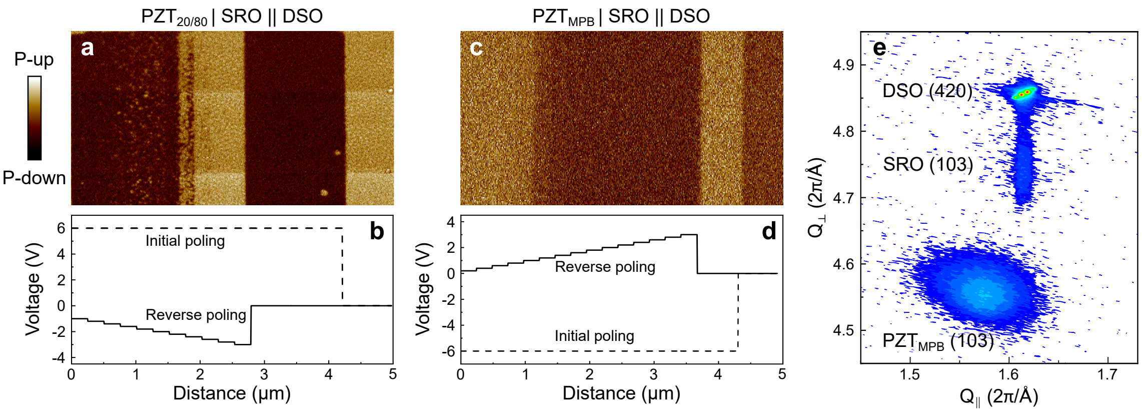


Figure S6. **Role of chemical composition and strain for multilevel polarization switching.** **a** vPFM image of a tetragonal ferroelectric $PbZr_{0.2}Ti_{0.8}O_3$ film on SRO-buffered $DyScO_3$ after applying the incremental reverse poling scheme specified in **b**. In the as-grown state the film is uniformly upwards polarization. The incremental reverse poling results in an abrupt nucleation of upwards polarized domains from the downwards poled region without any intermediate PFM signal levels. **c** vPFM image of a relaxed $PZT_{MPB}$ film on SRO-buffered $DyScO_3$ after applying the incremental reverse poling scheme specified in **d**. The film exhibits an pristine multidomain configuration without any intermediate vPFM signal levels upon incremental reverse poling. **e** Reciprocal space map around the $DyScO_3$ 420 peak revealing the strain-relaxed $PZT_{MPB}$ 103 reflection.

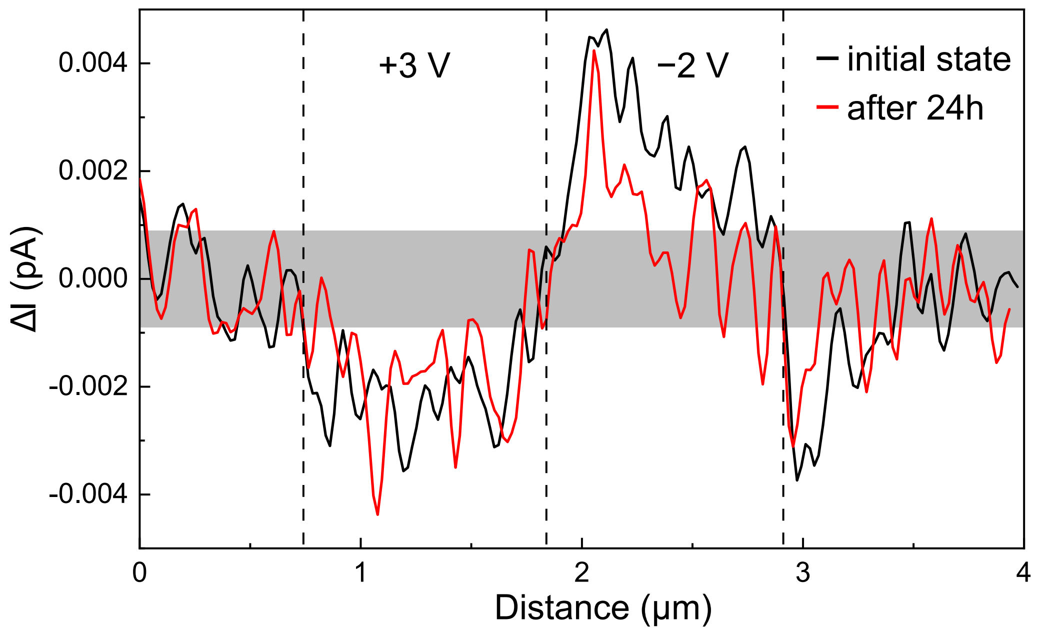


Figure S7. **Temporal stability of the polarization-modulated tunnel current measured with cAFM.** The current change with respect to the as-grown film ($\Delta I$) for two regions poled with $+3\,\mathrm{V}$ and $-2\,\mathrm{V}$ remains stable for at least 24 h. The gray area serves as a guide to the eye to show the current level of the as-grown film.


[1] Denev, S. A., Lummen, T. T. A., Barnes, E., Kumar, A. & Gopalan, V. Probing ferroelectrics using optical second harmonic generation. *J. Am. Cerm. Soc.* **94,** 2699-2727 (2011).
